# Potential of Phytase-Mediated Iron Release from Cereal-Based Foods: A Quantitative View

**DOI:** 10.3390/nu5083074

**Published:** 2013-08-02

**Authors:** Anne V. F. Nielsen, Inge Tetens, Anne S. Meyer

**Affiliations:** 1 Center for BioProcess Engineering, Department of Chemical and Biochemical Engineering, Technical University of Denmark, 2800 Kgs. Lyngby, Denmark; E-Mail: avfn@kt.dtu.dk; 2 Division of Nutrition, National Food Institute, Technical University of Denmark, 2860 Søborg, Denmark; E-Mail: intet@food.dtu.dk

**Keywords:** phytic acid, phytate, haem iron, non-haem iron, bioavailability, absorption

## Abstract

The major part of iron present in plant foods such as cereals is largely unavailable for direct absorption in humans due to complexation with the negatively charged phosphate groups of phytate (*myo*-inositol (1,2,3,4,5,6)-hexa*kis*phosphate). Human biology has not evolved an efficient mechanism to naturally release iron from iron phytate complexes. This narrative review will evaluate the quantitative significance of phytase-catalysed iron release from cereal foods. *In vivo* studies have shown how addition of microbially derived phytases to cereal-based foods has produced increased iron absorption via enzyme-catalysed dephosphorylation of phytate, indicating the potential of this strategy for preventing and treating iron deficiency anaemia. Despite the immense promise of this strategy and the prevalence of iron deficiency worldwide, the number of human studies elucidating the significance of phytase-mediated improvements in iron absorption and ultimately in iron status in particularly vulnerable groups is still low. A more detailed understanding of (1) the uptake mechanism for iron released from partially dephosphorylated phytate chelates, (2) the affinity of microbially derived phytases towards insoluble iron phytate complexes, and (3) the extent of phytate dephosphorylation required for iron release from inositol phosphates is warranted. Phytase-mediated iron release can improve iron absorption from plant foods. There is a need for development of innovative strategies to obtain better effects.

## 1. Introduction

Iron deficiency anaemia is the most common nutrition deficiency disorder worldwide and a problem in both developed and developing countries. Low iron intakes and poor iron absorption from the diet are common causes of anaemia with women of the child-bearing age, pregnant mothers, adolescents and the elderly being particularly susceptible [1]. Uptake of non-haem iron from plant foods is lower than that of haem iron from meat products. The main inhibitor of non-haem iron absorption in plant foods is phytic acid (*myo*-inositol (1,2,3,4,5,6)-hexa*kis*phosphoric acid). The phosphate groups of phytic acid are negatively charged under physiologically relevant conditions, resulting in phytate chelation of cations such as iron and zinc, making these minerals less available for absorption [2,3]. For vegetarians, elimination of meat coupled with high intakes of phytate-rich whole grains is known to lower iron absorption, increasing the risk of iron deficiency [4]. Analogously, consumption of the recommended daily intake of fibre-rich wheat bread has been found to impair iron status in young women with initially sufficient iron stores [5]. Phytases (mainly enzyme classes E.C. 3.1.3.8, E.C. 3.1.2.26) catalyse release of phosphate from phytate, in turn releasing the chelated minerals. Rather than relying on iron supplements or adding extra iron to foods, e.g., in the form of NaFeEDTA, phytase-catalysed dephosphorylation of naturally present iron phytate chelates in whole grain products is a potential option for increasing bioavailability of iron in the diet. This narrative review will examine the quantitative potential of phytase-catalysed iron release from plant-derived food components, with particular emphasis on the actions of the phytase catalysis *in vivo*. The text will mainly focus on the phytase-catalysed release of iron from phytate iron complexes present in cereal foods, since cereals and cereal-based meals represent the key plant based staple food type, in which phytate significantly retards or inhibits iron absorption.

## 2. Dietary Iron Bioavailability

Dietary iron generally exists in two forms: Haem iron and non-haem iron. Haem iron constitutes 50%–60% of iron in animal foods, whereas in plant foods the iron is found in non-haem form exclusively [6]. The average bioavailability (defined as the percentage absorption of the total amount of iron in the food) of haem iron is ~15%–35% [7], whereas non-haem iron is much less bioavailable, typically corresponding to ~1%–22% iron absorption [8,9,10,11], with cereal-based foods such as porridge having an iron absorption as low as 2%–3% [12].

The low bioavailability of non-haem iron is related to the chemical context of the iron in food. Non-haem iron in plant foods exists in a wide variety of chemical forms, e.g., ferric citrate, ferrous gluconate, ferrous fumarate, ferric dextrans, iron carbonyl, ferritin, ferric phytate, ferrous sulfate, ferrous carbonate, ferric chloride and ferric EDTA [13]. In wheat, the primary storage form of iron has for a long time been believed to be water-soluble monoferric phytate, constituting ~60% of the wheat bran iron [14,15]. Although a more recent study on wheat grains suggests that most of the iron in wheat is located in ferritin deposits [16] (a storage protein that contains up to 4500 solid mineral iron atoms per molecule [17]), the exact level of iron stored this way in wheat and perhaps, other cereals is not known at present. Ferritin iron seems to be readily bioavailable to humans, although the mechanism of absorption is still unclear [18,19].

Generally, iron in foods is in the oxidised ferric form (Fe^3+^), but if not adsorbed or bound to a storage protein, such as in haem iron or ferritin, the ferrous iron form (Fe^2+^) is considered to be the primary form absorbed by humans [13].

It is well established that complexing agents such as ascorbic acid, citric acid, other organic acids, as well as proteins (notably animal proteins from meat) and peptides enhance iron absorption [3,20,21,22]. Research conducted by García-Casal and co-workers suggests that also vitamin A and beta-carotene may enhance iron absorption [23,24]. On the other hand, phytate, tannins, phosphates, polyphenols and antacids inhibit iron absorption [25,26]. However, a fully validated algorithm for accurate prediction of iron bioavailability and absorption is not yet available [27], despite it being much needed, as well as for setting reference values for dietary iron intakes [28].

The absorption-enhancing effect of ascorbic acid is thought to be a result of the reducing properties of ascorbic acid, which reduce the less soluble Fe^3+^ ion to Fe^2+^ iron. Apart from being more readily absorbed, the Fe^2+^ form is also less prone to form complexes with polyphenols or phytate [29]. This redox theory is corroborated by the presence of a putative binding site for ascorbate on duodenal cytochrome-b (Dcyt-b, see Section 5 below on non-haem iron absorption), which catalyses the reduction of Fe^3+^ to Fe^2+^ prior to iron transport into the enterocyte [30]. Finally, ascorbic acid acts as a solubiliser, keeping iron in solution in the small intestine, thus enhancing absorption [31] and thereby, further preventing formation of the less soluble ferric phytate complexes [3].

Despite the findings that significant amounts of iron in wheat may be found in ferritin deposits [16], it is widely recognised that phytate is the major inhibitor of iron absorption from plant foods, notably in cereal-based diets [3,5,32,33,34]. It is not clear whether ferritin iron absorption is also inhibited by phytate, but available data suggests that the influence of phytate on ferritin iron absorption depends on the extent of ferritin degradation [35,36].

It has also been disputed to what extent iron status has an influence on how well haem and non-haem iron are absorbed, respectively, but the current consensus is that haem iron absorption is less affected than non-haem iron by iron status [29]. In any case, it is relevant to consider removal or degradation of phytate, *i.e.*, iron phytate complexes, as a means to improve iron absorption in iron-deficient individuals, particularly in those consuming high amounts of whole grain cereals.

## 3. Function, Structure and Properties of Phytic Acid

**Figure 1:**
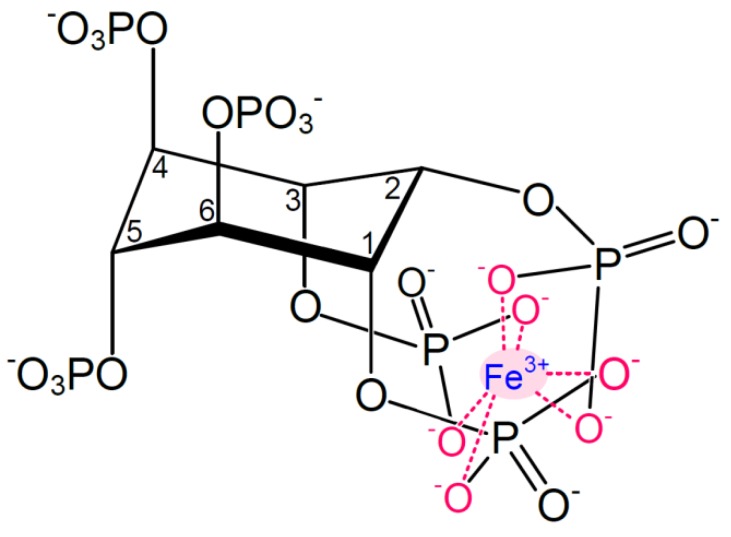
Structure of monoferric phytate, where Fe^3+^ is chelated via its six coordination sites. Adapted from [3].

Phytic acid (or phytate in the charged form) or *myo*-inositol (1,2,3,4,5,6)-hexa*kis*phosphate (Figure 1) is the phosphorous storage compound in plants, where it accounts for 60%–90% of the seed phosphorous [3,37].

During seed development, phytic acid is deposited as mixed phytate salts of potassium, magnesium, calcium, zinc and iron [38] in globoids; hence, several different cations may be associated with each phytate molecule. Around ~80% [3] of the globoids are localised in the aleurone layer of most cereal grains, e.g., wheat and barley, whereas in, for example, maize, the globoids are found in the embryo [2,39]. Wheat phytate globoids have been found to be constituted of proteins (46% w/w), phytic acid (40% w/w) and minerals in a concentration order of K (7.6% w/w) > Mg (3.2% w/w) > Ca (0.43% w/w) > Fe (0.059% w/w) [40]. At seed maturity, ~15%–20% of the iron in the grain is localised in the pericarp, ~70% in the endosperm and aleurone layer and 7%–8% in the embryo [41].

At physiologically relevant pH values (pH ~1.5–7), phytic acid is negatively charged, accounting for the ability of phytate to chelate mineral cations. The more phosphorylated the inositol rings, the stronger the interaction with iron and the lower the solubility. At physiological conditions, *i.e.*, in the intestinal environment, phytate thus forms complexes with ferric iron to form monoferric phytate (Figure 1). The pK_a_ values of the phosphate protons corresponding to the carbon numbering in Figure 1 are as follows: C1 + C3: 1.5 and 12.0, C2: 1.1 and 6.85, C4 + C6: 2.1 and 10.0, C5: 1.7 and 7.6 [42].

Monoferric phytate, which is the primary form of iron phytate, is water-soluble, but tetraferric phytate, *i.e.*, phytate chelating four Fe^3+^ ions is not, indicating that differences in bioavailability of iron from iron phytate complexes may be dependent on solubility of the different stoichiometric versions of iron phytate complexes [15].

Phytate is found in relatively high amounts in plant foods, particularly in cereals and legumes [3]. Daily intake of phytic acid varies largely according to diet from ~0.2–4.6 g globally, with, e.g., vegetarian diets generally containing higher amounts of phytic acid compared to mixed diets [3,43].

Reported values for iron and phytic acid content in various cereal foods vary widely, but typical values for phytic acid content are in the range of 0.5%–1% by weight (e.g., [3,43,44]) (Table 1). The wide range of phytic acid levels is related to methodology of phytic acid determination, plant variety and origin as well as how the plants have been cultivated [3]. Apart from influencing the phytic acid content, differences in growing conditions can also affect the iron content as well as the distribution of iron between phytate and ferritin in plants [13] and may thus significantly influence iron bioavailability of plant foods.

**Table 1:** Iron [45] and phytic acid (PA) content [43] in common cereal foods.

Food	Phytic acid (g/100 g)	Iron content (mg/100 g) ^1^	Estimated ^2^ molar ratio PA:Fe
Oatmeal	0.80–1.03	3.9	17–22
White bread	0.28–1.00	1.0	24–85
Whole grain bread	0.43–1.05	1.4	26–64
Rye bread (whole meal)	0.03–0.41	1.5	2–23
Rice	0.06–2.20	1.2	4–155

^1^ The iron contents represent mean values and were not measured in the same products as the phytic acid; ^2^ Rough estimates; calculated from a molar mass of phytic acid of 660 g/mol.

## 4. Iron Intake and Requirements

The iron contents of various cereal foods range from ~0.3–4 mg/100 g, with iron values in commonly consumed cereal foods ranging from ~1–4 mg/100 g (Table 1). For comparison, red meat contains ~0.2–2 mg iron per 100 g, but some meat products, e.g., liver, are very rich in iron, containing up to 31 mg iron per 100 g [45]. In order to fulfil the physiological requirements, approximately 1–2 mg iron must be absorbed per day depending on gender and age, with menstruating women and children requiring more iron than other population groups [46]. In common western diets, an average of around 10 mg ferric non-haem and 2 mg haem iron is consumed daily [25]. These levels match the recommended intakes for boys and men, that according to the Nordic Nutrition Recommendations, are 11 mg/day for boys aged 14–17, and 9 mg iron/day for men aged 18 and older [47]. However, for women of the childbearing age and lactating women, the recommendation for iron intake is 15 mg/day, with further recommendations on iron supplements during the second and third trimester for pregnant women [47], indicating that the iron requirements may not be met through the habitual diet.

## 5. Non-Haem Iron Absorption in Humans

Several factors determine the iron absorption from a meal: The amount and type of iron in the meal, the physiological mechanisms regulating iron absorption (uptake in intestinal cells and further transit) and the availability of the iron for uptake by the cellular transporters in the small intestine [48]. The iron status of the individual is another important determinant for iron absorption. Iron balance is maintained primarily by the iron-regulatory hormone hepcidin, which inhibits intestinal iron absorption and cellular iron release [25]. Hepcidin levels are upregulated by iron overload and inflammation, and downregulated by hypoxia and upon increased need for iron for erythropoiesis [25].

For the mechanism of absorption, haem iron is readily absorbed in the intestinal system by receptor-mediated endocytosis or direct haem transporters, although the detailed mechanisms are not fully elucidated [49].

More details are known regarding the mechanism for non-haem iron absorption. Non-haem iron is absorbed in the proximal part of the duodenum after reduction of Fe^3+^ (which is the more common oxidation state of iron in food) to Fe^2+^ by action of a ferrireductase, presumably duodenal cytochrome-b (Dcytb), present in the apical brush border of the intestinal wall [25,49]. A binding site for ascorbate on this enzyme underlines the role of ascorbic acid as an electron donor for Fe^3+^ reduction [30]. Fe^2+^ ions are then transported into the enterocyte using the divalent metal transporter-1 (DMT-1), which is also located in the apical brush border. This transport reaction is proton-driven, indicating that an acidic environment (optimal pH 5.5–5.8) is required for iron absorption, underlining that the primary site of iron absorption is just distal to the pylorus, where pH is still relatively low [25]. Expression of DMT-1 is induced in iron-deficient individuals [25]. Iron in the enterocytes can be retained or shuttled out of the cell, but the further fate of absorbed iron will not be considered in this review.

There is a surprising scarcity of data unravelling the details concerning the mechanism of cellular uptake of iron complexed to phytate and/or lower inositol phosphates (*i.e.*, inositols with ≤5 phosphates), but iron complexed with phytate is probably not directly bioavailable for entering the pathway for iron absorption described above. The currently available evidence is thus weak or non-existent, but a plausible scenario is that absorption of iron bound in iron phytate complexes into the enterocytes from the duodenum lumen can only occur after the iron has been released from the phytate, and reduced from the Fe^3+^ to the Fe^2+^ form. It remains to be elucidated, whether and how Fe^3+^ more loosely chelated to inositol phosphates is absorbed. Competition between ascorbic acid and phytate as iron chelators may play a role [3], as the ascorbic acid-iron complex is presumably available for absorption.

## 6. Chemical Form of Iron Phytate Complexes in the Gastrointestinal System

The chemical form of the phytate in the gut is an important parameter for the assessment of non-haem iron bioavailability in the gut. Phytate shows increasing chelating affinity, *i.e.*, lower dissociation constant, from mono- to multivalent cations [3]. However, factors such as competing chelating agents should also be taken into consideration when evaluating the iron phytate interactions in the gut.

As previously mentioned, the pK_a_ values of the phytate phosphates result in phytate being negatively charged at physiologically relevant pH values, *i.e.*, pH values lower than ~1 are necessary for phytic acid to be in the fully protonated form. After a meal, pH in the stomach is typically ~3–7, decreasing gradually over a few hours to the fasting pH ~2 [50,51,52]. Assuming that the pK_a_ values are independent of iron binding, phytate will thus carry six negative charges in the gastric ventricle (pH range ~3–7).

It has recently been experimentally confirmed that the iron phytate complex will be primarily in the associated monoferric form in the pH range above ~4, with Fe^3+^ existing as free ferric ions or ferric hydroxides at lower pH values [53]. The predominating forms between pH 3 and 7 are tri-, di-, mono- and non-protonated monoferric phytate with non-protonated monoferric phytate being the prevailing form at pH values exceeding ~5.8 [53] (Figure 1).

Studies on the sequestering ability of phytate have shown that the relative affinity of phytate towards different metal cations depends on pH, meaning that for example at pH ~6–8, Fe^3+^ is chelated more strongly than Cr^3+^ and Al^3+^ ions, whereas iron is the least strongly chelated of the three metal species at pH values below ~6 [53]. Furthermore, the concentration of phytate at which 50% of iron present in trace amounts is chelated has been found to range from 4.7 × 10^−6^ M to 1.1 × 10^−10^ M from pH 4.0–7.4 [53]. Hence, if, for example, 60 g oatmeal (containing, e.g., 0.9 g phytic acid/100 g, see Table 1) is consumed with milk resulting in a total volume of the gastric ventricle after intake of ~0.5 L, the potential concentration of phytate in the stomach is ~1 mM, thus at least 1000-fold higher than the concentration necessary to sequester 50% of the iron ions. Ismail-Beigi *et al*. [54] found that wheat bran binds 72% of iron (0.5 mg/L) *in vitro* at pH 6.5–6.8 (duodenal pH range), which corroborates that most iron is associated with phytate in the pH conditions of the human gastrointestinal system.

Furthermore, it has been proposed that inositol triphosphate (*myo*-inositol phosphorylated on C1, C2 and C3) quantitatively chelates Fe^3+^ in a 1:1 ratio at physiologically relevant pH values (including acidic environments) [55]. Another study has shown that complexation of phytate with Fe^3+^ causes acidification, meaning that phytate is further deprotonated upon association with Fe^3+^ and that at Fe^3+^:phytate ratios higher than ~4, intermolecular interactions will occur, forming a network of ferric phytates causing precipitation of aggregates [56].

As earlier mentioned, phytate in the plants naturally exists as mixed salts of several different mineral cations. It can therefore be speculated that under physiological conditions in the gastrointestinal system, the phytate molecule will still be associated with several different cations as well as the iron, although no exact information could be found on this matter.

## 7. Degradation of Phytate in the Human Gastrointestinal System

Very few studies have been conducted on the hydrolysis of phytate, notably iron phytate complexes and phytate globoids, in the human gastrointestinal system, including the hydrolysis of phytate in the stomach [57]. Early studies showed only limited degradation, *i.e.*, dephosphorylation, of phytate in the human gastrointestinal system as measured by recovery of ~40%–70% phytate in faeces [58,59].

Human ileostomy studies conducted by Sandberg’s group [60,61,62] showed that 95% ± 10% of phytate initially present in the diet was recovered after passage of an extruded wheat bran diet through the stomach and small intestine with no detection of lower inositol phosphates, whereas 42% ± 12% of the phytate was recovered in an unprocessed wheat bran diet and with detectable amounts of inositol penta-, tetra- and triphosphates in the ileostomy content [62]. They also found that the absorption of Zn, Mg and P was significantly decreased from the extruded product, whereas absorption of Ca and Fe was at the same level for the two products, indicating that phytate needs to be dephosphorylated to a higher extent for Fe and Ca release to occur compared to Zn, Mg and P.

It was later shown that very low phytase enzyme activity (see Section 8 on phytases below) is present in the human small intestine [63], so degradation of phytate in the gastrointestinal system is most likely ascribable to the presence of dietary phytase enzyme (endogenous to wheat) [64], although it has later been suggested that after the initiation of phytate dephosphorylation by endogenous food phytases, intestinal phosphatase enzymes may play a role in continuing the dephosphorylation [57].

As iron is absorbed in the small intestine, phytate hydrolysis occurring after this point in the intestinal system is not relevant with regard to increasing iron absorption. Therefore, phytate hydrolysis in the large intestine is not considered in the present review.

In conclusion, natural degradation of phytate in the human gastrointestinal system is insufficient with regard to the release of absorbable iron from iron phytate complexes. One strategy that has been suggested to alleviate this problem is the use of exogenous phytase enzymes, catalysing the dephosphorylation of phytate, in food processing and during *in vivo* digestion.

## 8. Phytases

Phytases are a subgroup of phosphorolytic enzymes that are capable of initiating (and continuing) the hydrolysis of phosphate groups from phytate. Phytases are used widely in feed for non-ruminant animals [65] and are classified according to their catalytic mechanism (histidine acid phytases, β-propeller phytases, cysteine phytases and purple acid phytases), pH optima (acid or alkaline phytases) and site of phytate hydrolysis initiation (3-phytases, E.C. 3.1.3.8; 6-phytases, E.C.3.1.3.26 and 5-phytases, E.C.3.1.3.72) [66].

Most commercially available feed phytases are of fungal (*Aspergillus niger*) or bacterial (*Escherichia coli*) origin; they include both 3- and 6-phytase type histidine acid phytases and have a molecular weight of ~45–50 kDa, pH optima range within ~2.0–5.5 and temperature optima at ~50–60 °C [65]. Many others have reviewed the properties, reaction mechanisms and production of different phytases, see, e.g., [2,65,66,67,68,69,70].

The World Health Organization has recently evaluated the 3-phytase from *Aspergillus niger* for use in and with food for humans and found it safe for consumption with an acceptable daily intake “not specified” [71]. It remains to be seen whether other phytases that are used for animal feeds will be recognised as safe for human consumption.

On this note, a patent for an iron fortification nutritional blend containing phytase has recently been granted to DSM, corroborating the industrial potential and actuality of these enzymes for defying iron deficiency in humans [72].

## 9. Degradation of Iron Phytate Complexes in the Gastrointestinal System

### 9.1. Activity of Phytases in the Gastrointestinal System

The gastrointestinal system constitutes a hostile environment for most enzymes, particularly due to the low pH in the stomach and the presence of proteolytic enzymes, notably pepsin in the stomach and trypsin and chymotrypsin in the small intestine.

It has been shown that endogenous wheat phytases are mostly active in the gastric ventricle, where they retain ~9% of their activity (compared to the activity in the feed of 43 mU/mg protein), whereas in the small intestine activity retention is only ~2%; in practice meaning that wheat phytase-catalysed phytate degradation takes place in the stomach only [73].

The enzyme inactivation is caused by proteolysis by pepsin in the pylorus and unfavourable pH conditions in the duodenal chyme (pH 6.5–7), as wheat phytase has a pH optimum of 5.5 and 6.0 [40].

For comparison, *A. niger* phytase is expected to retain 50%–60% activity in the stomach [74] and has optimum activity at pH 2.0 and 6.0 at 37 °C with activity in the entire range from pH 1.0–7.5 [75].

In one study, *A. niger* phytase has been found to continue phytate degradation in the duodenum [76]. Another study reported that *A. niger* phytase retained 95% activity after incubation at pH 3.5 with 5 mg pepsin/mL compared to only 70% activity retention for the wheat phytase [77]. *E. coli* phytase has been found to exhibit an even higher stability in an acidic proteolytic (pepsin) environment compared to the *A. niger* phytase, whereas *A. niger* phytase has been reported to be more stable than *E. coli* phytase when incubated with trypsin at pH 7.5 [68]. On a final stability note, fungal phytases generally have higher thermal stability than bacterial phytases [65].

### 9.2. Role of Exogenous Phytases in Gastrointestinal Phytate Degradation

Supplementation of exogenous *A. niger* phytase to a wheat-based meal can improve iron absorption compared to endogenous wheat phytases by degradation of phytate in the gastrointestinal system [75]. Unfortunately, data from human studies regarding the details and mechanism of degradation of phytate (notably iron phytate and phytate globoids) in the gastrointestinal system using exogenous phytases are not available. However, an *in vivo* study in pigs has shown that endogenous feed phytases as well as added *A. niger* phytase (1800 FTU/kg feed) can catalyse the hydrolysis of phytate. This hydrolysis seems to be dependent on the extent of phytate solubility in the stomach (which was ~2/3) [73], as higher dosage of exogenous phytases did not increase phytate degradation. This indicates that solubility rather than catalytic activity limited phytate degradation. However, degradation of phytate to lower inositol phosphates increases their solubility [73] and susceptibility towards further degradation and lowers their affinity for mineral chelation [3,78].

Due to the pH increase, solubility of the inositol phosphates decreases as the food chyme is passed on to the duodenum, with lower inositol phosphates still exhibiting higher solubility than more phosphorylated inositols [3,73]. As the higher inositol phosphates have a higher tendency towards mineral chelation, the result is co-precipitation of mineral ions, e.g., Fe, Zn and Ca, thus making them unavailable for absorption [3]. On the other hand, the lower inositol phosphates, notably inositol di- and triphosphates, seem to keep the minerals in solution in the small intestine and potentially aid in the absorption of minerals in the small intestine [3].

Further hydrolysis in the duodenum is limited by the low solubility of the iron inositol phosphate complexes [73] as well as the acidic pH optima of most of the available microbial phytases that are under consideration for this application. Low substrate solubility in the small intestine is the main reason why the gastric ventricle is considered the primary target site for action of exogenous phytases for both phosphate (particularly relevant in animal nutrition) and iron release from iron phytate complexes.

### 9.3. Extent of Phytate Degradation and Iron Absorption

Having phytate partly dephosphorylated, the question is to which extent dephosphorylation is required to release iron for absorption. In an early study, lower inositol phosphates, notably inositol hexa- to tri-phosphates, were found to impair iron absorption proportionally to the degree of phosphorylation [79]. In a subsequent study conducted by the same group it was found that inositol hexa- and pentaphosphates inhibit iron absorption, whereas inositol tetra- and triphosphates alone do not inhibit iron absorption due to lower affinity for Fe^3+^ chelation [80]. However, the inositol tetra- and triphosphates were found to interact with higher inositol phosphates in inhibiting iron absorption and thus, it was concluded that at least four phosphates should be hydrolysed from phytate in order to significantly improve iron absorption from cereals and legumes.

Quantitatively, Yu and co-workers found that, compared to inositol hexaphosphate, the inositol pentaphosphates had a chelating power of ~70%–76%, inositol tetraphosphates ~35% and inositol triphosphates ~30%, whereas inositol di- and monophosphates exhibited no chelating power towards Fe^3+^ at pH 2.4 [78]. The stoichiometry of the detected iron phytate complex under these conditions was 2:1 [78].

In order to significantly improve iron bioavailability, complete degradation of phytate is therefore recommended [12,57]. Complete phytate degradation, *i.e.*, complete dephosphorylation, is expected to increase iron absorption five-fold or more [12] (up to 12-fold [34]), whereas a ~90% degradation would cause only a two-fold increase in iron absorption [12]. Lower degrees of hydrolysis are not expected to be useful with regard to iron absorption, since the iron will still be bound to phytate or lower inositol phosphates, but if complete dephosphorylation of phytate cannot be achieved, it is advised to keep the molar phytate:iron ratio below ~1:1, preferably below ~0.4:1, in order to limit the phytate chelation of iron that renders the iron unavailable for absorption [34].

## 10. Potential of Phytase-Mediated Iron Release from Plant Foods

When assessing the potential of phytase-mediated iron release from plant foods in human studies, several issues need to be considered, e.g., iron status of the individuals in the study; dietary factors including, but not limited to, phytate content; how iron status can be assessed (which parameters are more suitable for evaluation and how informative *in vitro* studies can be conducted); where the phytate hydrolysis should take place (e.g., prior to ingestion or during digestion); to which extent the phytate should be dephosphorylated to significantly release iron; in which form released iron is present and the bioavailability of this iron form.

When considering *in vivo* phytase catalysis, a range of questions arises regarding where in the gastrointestinal system and to what extent the phytase is active, what the form of the substrate (soluble, insoluble/aggregated, dissociated or complexed with minerals, degree of protonation) is at the site of catalysis and the affinity of the phytase for this form of phytate. It has, for instance, been shown by Tang *et al.* [81] that if phytate is present as precipitated phytate salts (e.g., ferric phytate) at pH 6, phytases from neither wheat *Aspergillus ficuum* nor *Bacillus subtilis* are able to catalyse the dephosphorylation of phytate. On the other hand, Bohn *et al.* [40] found that wheat phytase was active on phytate globoids at pH 6, indicating that the natural form of the phytate is accessible for degradation.

When phytate is degraded *ex vivo* prior to ingestion of the food, the process can be monitored more easily and more reproducible and predictable results are likely to be obtained.

With regard to the released iron, whether it is released prior to or after consumption, it is relevant to know its chemical form and biological availability.

### 10.1. Effect of Evaluation Parameters in *in Vitro* Studies

Generally, three parameters are most often assessed when results from *in vitro*/*ex vivo* studies are reported:

1. Extent of phytate degradation and content of lower inositol phosphates;
2. Dialysability of iron;
3. Cellular availability and/or uptake of iron.

Of these, the last is the pertinent health parameter and the question is therefore to what extent the cellular availability and uptake of iron is explained by the other parameters, which are usually easier to evaluate.

#### 10.1.1. Phytate Degradation

Phytate degradation is one of the most reported parameters, and possibly the least relevant of the three. In assessing reported results on phytate degradation, it is important to consider the method of phytate determination as different methods will report different results. Two methods exploiting the interaction of ferric iron with inositol phosphates are used, either for precipitation of ferric phytate (originally developed by Heubner and Stadler [82]) or as interactions resulting in soluble complexes as is the case with Wade’s reagent (ferric chloride and sulfosalicylic acid) [83]. These are relatively non-specific for phytate, as lower inositol phosphates are also able to participate in these interactions. Confirming this, Sandberg and co-workers concluded that the iron precipitation method was not suitable for evaluating phytate degradation [62]. Instead, various chromatographic separations can be performed to separate phytate from lower (*i.e.*, less phosphorylated) inositol phosphates and thus give a better picture of the degree of dephosphorylation (e.g., [84]). Due to the significantly different outcome resulting from the use of different methods, some of the quantitative comparisons of the results obtained in different studies (regarding phytate degradation) are hampered by the influence of different methodologies. In particular, this influence hampers the complete understanding and comparisons with respect to the possible significance of inositols phosphorylated to different extents on the release and absorption of iron.

#### 10.1.2. Dialysability of Iron

*In vitro* dialysability studies are useful in screening experiments when comparing iron absorption between different food items or meals, but a dialysis may exclude iron bound in large complexes that are bioavailable and on the other hand may include iron bound in smaller complexes (e.g., lower inositol phosphates and even phytate [85]) and yet are not bioavailable [86]. Argyri and co-workers found a good correlation between iron dialysability (particularly ferrous iron dialysability) and iron absorption in humans, indicating that ferrous iron dialysability can explain ~75% of the variation in *in vivo* absorption studies (67% explained by total dialyzable iron) [87]. Others report that dialysis studies usually, but not always, predict the right direction of the response (iron absorption) and that the magnitude of the response does not always correlate with what is found in human studies [88].

#### 10.1.3. Caco-2 Cellular Iron Uptake

Even though there are no substitutes for *in vivo* studies, Caco-2 cell models provide an alternative to expensive and time-consuming human studies [89] and it has been demonstrated that Caco-2 cells models can correctly predict the influence on iron bioavailability response of all key iron absorption modifiers [88]. *In vitro* model systems with Caco-2 cells are normally implemented with a two-phased simulated digestion mimicking the conditions in the stomach and the small intestine [90]. Often these simulations are run with a pH ~2 phase simulating the gastric ventricle and a pH ~7 phase simulating the small intestine. This model, however, has a risk of overestimating the phytate degradation due to the very low pH value in the stomach simulation. Studies have shown that pH in the stomach after a meal is in the range of ~3–7, meaning that phytate complexes will be less soluble *in vivo* than in the *in vitro* model, demonstrated by the finding that ~5% of phytate is soluble at pH 5.5 in simulated wheat gruel digesta compared to ~55%–70% at pH 2–4 [91]. The pH-dependence of solubility poses a potential bias of many *in vitro* studies, which should be kept in mind when evaluating results from these studies.

Iron uptake in Caco-2 cells can be measured either as incorporation of radiolabelled iron or as an increase in ferritin in the cell, both of which have shown good correlation with iron absorption in humans from some *in vivo* studies on the effect of different dietary factors, whereas for other studies, no correlation has been found [88,90,92]. With regard to the influence of polyphenols and phytate on iron uptake, it has been reported that the sensitivity of Caco-2 cells models is similar to what is observed in human studies [88].

When compared to the dialysis evaluation, cellular uptake provides a better estimate of *in vivo* absorption, as dialysis only evaluates passive diffusion of iron, whereas in cell models, active cellular iron absorption is also taken into consideration [85]. It is also possible that the total fraction of dialysable iron is not available for cellular absorption [85], indicated, e.g., by a lack of proportionality between iron dialysability and Caco-2 cellular uptake in a study by Sanz-Penella *et al*., where it was explained by inhibitory concentrations of inositol hexa- and pentaphosphates [93]. Lynch concludes that the Caco-2 cell model may not accurately reflect the magnitude of the effects that influence iron absorption [48] and it should also be kept in mind that Caco-2 cell studies are often conducted with one layer of cells, thus having a significantly lower surface area available for absorption compared to the *in vivo* intestinal system [93].

### 10.2. Effect Evaluation Parameters in *in Vivo* Studies

Iron bioavailability from foods is often evaluated by quantifying uptake of radio- or stable isotope labelled extrinsic iron that has been added to the food [89]. Otherwise, general iron status and iron absorption in *in vivo* studies can be evaluated using iron status indicators such as blood haemoglobin concentration, serum ferritin, transferrin receptors and/or iron concentration. Each of these has advantages and disadvantages: Haemoglobin concentration is a measure of anaemia and does not necessarily evaluate iron status, as iron deficiency and anaemia do not always occur concomitantly [94]. Also, normal haemoglobin ranges varies widely among individuals, making assessment of general iron deficiency difficult and they may also be affected by other factors such as malaria, malnutrition, age and pregnancy [95]. Serum ferritin has been shown to be a good indicator of body iron stores in healthy individuals, but is influenced by infections and acute or chronic inflammations [96]. Transferrin receptors are carrier proteins for transferrin and used as a marker of iron status. Transferrin receptors as an iron indicator has the advantage over serum ferritin that it is unaffected by inflammatory conditions [97]. Serum iron is linked to iron adequacy for development of red blood cells, but is not a good indicator of iron status due to diurnal and post-meal variations [94].

### 10.3. Previous Studies on Phytases for Food Processing *in Vitro* and *in Vivo*

#### 10.3.1. Studies Evaluating Efficacy of *in Vivo* Phytase Catalysis

Some of the earliest studies assessing the potential of enzymatic phytate degradation *in vivo* in humans were conducted by Sandberg and co-workers, who already in 1988, in a meal-study over two four day periods, showed that addition of extra endogenous wheat phytase in the form of bran increased phytate degradation *in vivo* compared with addition of phytase-deactivated bran [64] (Table 2). The potential of exogenous phytases for improving iron bioavailability was demonstrated by this group by assessing iron absorption from wheat rolls containing added fungal phytase (*Aspergillus niger* phytase). The iron absorption increased 83% from 14.3% to 26.1% [75] (Table 2). A more recent study has also shown that addition of *A. niger* phytase to cereal porridge can increase iron absorption 23%–70% (with a better effect on iron as FeSO_4_ compared to NaFeEDTA) or up to 75% when added together with ascorbic acid [74] (Table 2). The study reported a total effect of ascorbic acid and phytase of ~400% increase in iron absorption from NaFeEDTA compared with iron absorption from FeSO_4_ given without absorption enhancers [74] (Table 2). This is in line with the consensus that ascorbic acid is a non-haem iron absorption enhancer [25].

All studies designed to boost *in vivo* phytate degradation via addition of *A. niger* phytase to a meal (with the enzyme added just prior to the ingestion, e.g., drizzled on top of bread or mixed into porridge) have shown that the phytase can act *in vivo* and generate a positive effect on iron bioavailability (Table 2). To our knowledge, no studies have documented the effect of *in vivo* phytate hydrolysis in humans catalysed by phytases from other organisms, but as discussed below, other phytases have been shown *in vitro* to potentially exhibit effects similar to the *A. niger* phytase. Iron absorption in response to phytase addition in the reported *in vivo* studies have ranged from 2.4% to 26% and with iron intakes per meal ranging from ~2.5 to 8 mg. This increased iron absorption in effect results in absolute iron absorptions of up to ~0.5–1 mg per meal. Such an improvement infers that a cereal-based meal with phytase-enhanced iron absorption can produce a significant improvement with regard to the average requirements of 1–2 mg iron/day.

**Table 2:** Condensed results from *in vivo* phytase catalysis studies. Abbreviations: CA: citric acid; AA: ascorbic acid; AN: *Aspergillus niger*; M: male; F: female; C: children; ID: iron-deficient; NA: non-anaemic; SF: serum ferritin; Hb: haemoglobin; CRP: C-reactive protein; TfR: transferrin receptor; w/wo: with/without.

Treatment	Study design and duration	Iron dosage	Test group	Evaluation parameter	Result	Reference
Active or inactivated endogenous wheat bran phytase	Meal study 2 × 4 day periods	n/a	8 M, 1 F ileostomy patients (no information on iron status)	Phytate degradation (Fe absorption not evaluated)	↑ from ~5% with deactivated wheat bran phytase to ~60% with active wheat bran phytase	[64]
Active or inactivated endogenous wheat phytase or AN phytase	2 separate meal studies with wheat rolls: (1) Active or inactivated wheat phytase; (2) Phytase-inactivated wheat bran w/wo AN phytase	3.7 mg labelled Fe + 0.4 mg intrinsic Fe/meal	9 M, 11 F split into 10 in each substudy (no information on iron status)	Fe absorption (measurement of radiolabelled ^55^Fe/^59^Fe in blood samples)	(1) No difference; (2) ↑ 83% from 14.3% ± 2.6% to 26.1% ± 3.8% with AN phytase	[75]
Maize porridge taken with different micronutrient powders containing Fe (as NaFeEDTA or FeSO_4_) with AN phytase, AA, l-α-glycerophospho-choline and/or Ca	Meal study Crossover design (2 days)	3 mg Fe/meal	101 F (21 ID, 1 ID anaemic) allocated to 6 separate iron absorption studies (*n* = 16–18 in each substudy)	Fe absorption (stable isotope labelled ^57^Fe/^58^Fe measurement in erythrocytes 14 days later)	*Phytase*: ↑ 23%–75% from 2.4%–5.0% to 4.1%–7.4%. Highest effect of phytase was observed in combination with ascorbic acid, where total increase as result of phytase, ascorbic acid, and NaFeEDTA resulted in 400% absorption increase compared to iron absorption from FeSO_4_ (1.5% to 7.4%). No significant effect of other single factors	[74]
High-phytate porridge taken with micronutrient powder containing Zn, Fe as NaFeEDTA, AA and AN phytase	Diet study (5 days/week for 23 weeks) Double-blind controlled study Control: No Fe supplement	2.5 mg Fe/meal	200 C (low iron status)	Fe status (SF, Hb, CRP, TfR content in blood samples)	Occurrence of Fe deficiency ↓ 75% (↓ 35% in control group) Body Fe ↑ 100% (↑ 40% in control) Fe absorption ~7%–8%	[98]
Fe-rich bread w/wo phytase supplement compared with FeSO_4_ supplement	Meal study	5–8 mg Fe/meal	24 F (borderline anaemic) allocated to 5 test meals	Fe status (serum iron measured 180 and 210 min after ingestion)	Serum Fe ↓ in all groups at 180 and 210 min after intake except for the positive control group given FeSO_4_ supplement. Decrease in serum Fe was largest in the order of iron-rich bread > control bread > iron-rich bread with 0.010% (w/w) phytase > iron-rich bread with 0.015% (w/w) phytase	[99]

One research group observed a decline in serum iron after the study subjects had ingested the iron-rich meals [99]. Such a decline was also observed in a study conducted by Conway *et al*. [100], where the serum iron decline correlated with high amounts of phytate in the ingested meal. However, phytase diminished the serum iron decline compared to the control meal in the study by Bokhari *et al*. [99], indicating that high phytate intake could explain the decrease in serum iron levels, although in this latter study, the phytate content was unfortunately not reported. One study did not include a control group that received the meal with iron supplement, but without added phytase (although they did have a control group receiving a meal without any iron supplement), making it difficult to single out the added effect of the phytase on the iron absorption [98].

The available studies are obviously quite different with regard to design of study, target group, compositions of the meals or whole diets, especially with regard to the content of phytase and phytate:iron ratio, making any firm conclusions difficult. However, overall, the available data suggest that *A. niger* phytase may have the potential to improve *in vivo* iron absorption in humans.

#### 10.3.2. Studies Evaluating Efficacy of *ex Vivo* Phytase Catalysis on *in Vivo* Parameters

Effect of phytase-mediated pretreatment of cereal-based foods has been documented as increases in *in vivo* iron absorption from 78% up to as much as 1066% in single meal studies [12,101] (Table 3). However, a four-month intervention study with 41 women did not show an effect of phytase pretreatment [5] (Table 3). This was probably due to the low degree of phytate dephosphorylation (~22%) achieved with the enzyme treatment in this study, which was not enough to release iron from the phytate complexes (InsP5+6:Fe ratio was ~0.6 in the phytase-treated bread).

#### 10.3.3. Studies Evaluating Efficacy of *ex Vivo* Phytase Catalysis on *in Vitro* Parameters

*In vitro* studies have shown that *A. niger* phytase has the potential to degrade phytate and thereby improve potential for iron absorption during *in vitro* digestion or as a pretreatment step for foods prior to ingestion (Table 4). One study found phytate degradation in bread with *A. niger* phytase to be up to 97%, resulting in an increased cellular iron uptake by Caco-2 cells of ~150% [102]. Similar results were obtained by Sanz-Penella *et al*. [93] (~97% InsP5+6 degradation, 92% increase in iron uptake by Caco-2 cells) (Table 4).

Whereas reasonable correlation was seen between Caco-2 cell iron uptake and phytate degradation, the correlation between phytate degradation and dialysability is unclear, see e.g., Sanz-Penella *et al*. [103] and Akhter *et al*. [104], who find very different correlations between phytate degradation and iron dialysability (Table 4). This indicates that inositol phosphates (and especially lower inositol phosphates) can be dialysed, but are not available for cell uptake. The finding that iron uptake by Caco-2 cells only increase after extensive phytate degradation, *i.e.*, in the order of >90% degradation, also underlines that phytate degradation needs to be virtually complete and phytate:iron ratios should be low for iron absorption to increase [34,57]. One group has also shown potential of other microbial phytases (*E. coli*, *A. fumigatus*) for phytate degradation [21] (Table 4).

**Table 3:** Condensed results from studies employing *ex vivo* phytase catalysis evaluated on *in vivo* parameters. Abbreviations: CA: citric acid; AN: *Aspergillus niger*; M: male; F: female; C: children; ID: iron deficient; NA: non-anaemic; SF: serum ferritin; InsP6: phytate; InsP3: *myo*-inositol triphosphate.

Treatment	Study design and duration	Iron dosage	Test group	Evaluation parameter	Result	Reference
AN phytase addition to cereal porridges during manufacturing	Meal study	2.5 mg Fe/meal	34 M (1 ID), 44 F (13 ID), all NA	Fe absorption (measurement of radiolabel-led ^55^Fe/^59^Fe in blood samples)	↑ 209%–1066% from 0.3% to 2.4%→2.8%–11.5%	[105]
AN phytase added during making of fibre-rich wheat bread	Intervention study; 4 months	6 mg Fe/meal, 14 mg/day	41 iron-sufficient F	Fe absorption *in vivo* (SF) InsP3-6 content in bread	No effect on SF by phytase treatment; InsP5+6 contents ↓ 17%; InsP3-6 content ↓ 22%	[5]
CA and AN phytase addition to oat beverage as pretreatment	Meal study, 4 days	1.3 mg Fe/meal	23 M, 22 F (non-ID)	Fe absorption *in vivo* (radio-labelled ^55^Fe/^59^Fe count in whole body and eryth-rocytes); InsP3-6 content in oat beverage	CA: ↑54% (from 3.9% to 6.0%) Phytase added with CA further increased iron absorption by 78% (6.0%→10.7%); Phytase reduced InsP3-6 to undetectable amounts (<1 mg/portion), phytate-P ↓ 83%	[101]

**Table 4:** Condensed results from studies employing *ex vivo* phytase catalysis evaluated on *in vitro* parameters. Abbreviations: CA: citric acid; AA: ascorbic acid; AN: *Aspergillus niger*; InsP: *myo*-inositol phosphate; InsP6: phytate; InsP5: *myo*-inositol pentaphosphate; InsP3: *myo*-inositol triphosphate; w/wo: with/without.

Treatment	Evaluation parameter	Result	Reference
Addition of AN, *A. fumigatus* or *E. coli* phytase w/wo CA and AA to whole-wheat bread dough	Phytate ^1^ degradation Fe dialysability from bread	*CA*: Phytate degradation ↑ from 42% in control to 69%; Fe dialysability ↑ 12-fold *AN phytase alone*: Phytate degradation 57%, no significant effect on dialysability *Microbial phytases* + *CA*: *AN*: Phytate degradation 74%–85% (dose-dependent); Fe dialysability ↑ 15-fold. *E. coli*: Phytate degradation 63%–76% (dose-dependent); Fe dialysability not assessed *A. fumigatus*: Phytate degradation 83%–85% (dose-dependent); Fe dialysability not assessed *AN phytase* + *CA* + *AA*: Fe dialysability ↑ 24-fold compared to control	[21]
AN phytase addition during making of different bread	Phytate ^1^ degradation	Phytate degradation ↑ 12%–57% compared to control depending on bread type, total phytate degradation with phytase: 49%–90%	[106]
High-phytase producing yeast strains addition during *in vitro* digestion of wheat gruel	Phytate degradation	≤59%	[91]
Bifidobacteria cell suspensions or AN phytase addition during wheat bread making	InsP3-6 content in bread	*Phytase*: each InsP ↓ 67%–100% (mean ± standard deviation 91% ± 10%) compared to control; *Bifidobacteria*: each ↓ −8%–67% (19% ± 27%) compared to control	[107]
Highly phytase-producing yeasts (experimental strains), *S. cerevisiae* or *A. ficuum* phytase addition to togwa prior to ingestion	Phytate degradation after 48 h fermentation	*S. cerevisiae*: 85% *A. ficuum* phytase: 89% *Experimental strains*: 95% *Control*: 51%	[108]
Activating endogenous phytase in fortified and non-fortified flour/oilseed mixtures	Fe dialysability	*Wheat/soy*: ↑ 43%–162% (non-fortified); ↑ 40%–168% (fortified); *Wheat/groundnut*: ↑ 83%–192% (non-fortified); 97%–240% (fortified) Dialysable Fe in controls: 1.3%–9.7%	[109]
AN phytase addition during *in vitro* digestion of bread	Phytate degradation Fe bioavailability in Caco-2 cell model	Phytate degradation 89%–97% Cellular uptake of Fe ↑ 152%–156%	[102]
Fungal phytase or phytase-producing bifidobacteria addition during whole wheat bread- or whole wheat sourbread-making	InsP5 + InsP6 degradation Fe dialysability Ferritin formation in Caco-2 cells	*Whole wheat bread*: *Fungal phytase*: InsP5+6 degradation ~97%; dialysable Fe ↑ 17%, dialysability ↓ 9%; Caco-2 cell uptake ↑ 92% *Bifidobacteria*: InsP5+6 degradation ~76%; dialysable Fe ↑ 127%, dialysability ↑ 118%; Caco-2 cell uptake ↑ 33% (not significant) *Whole wheat sourbread*: *Fungal phytase*: InsP5+6 degradation ~68%; dialysable Fe ↑ 45%, dialysability ↑ 82%; Caco-2 cell uptake ↑ 67% (not significant) *Bifidobacteria*: InsP5+6 degradation of ~58%–70%; dialysable Fe ↑ 283%–456%; dialysability ↑ 236%–391%; Caco-2 cell uptake ↑ 0%–25% (not significant)	[93]
*A. oryzae* phytase or phytase-producing bifidobacteria addition during pretreatment of infant cereals	Phytate degradation Fe dialysability	*A. oryzae phytase*: InsP6 ↓ 45%–67% compared to control; InsP3-6 (total) ↓ 25%–28%; no change in Fe dialysability *Bifidobacteria*: InsP6 ↓ 75%–87% compared to control; InsP3-6 (total) ↓ 23%–63%; no change in Fe dialysability	[103]
Exogenous wheat phytase addition to wheat flour	Phytate ^1^ degradation Fe dialysability	Phytate ↓ 35%–69% Fe dialysability ↑ 11%–52% (from 12% to 16% to 15%–25%)	[104]

^1^ The term phytate has here been used to cover the higher inositol phosphates (as determined by Wade reagent or precipitation).

## 11. Other Options for Enhancing Iron Absorption

Other options for enhancing iron bioavailability from meals and/or whole diets have been reviewed extensively elsewhere (e.g., [3,34,110]) and includes the pretreatment of grains, e.g., soaking, malting and germination, as well as addition of organic acids, in particular ascorbic acid and citric acid, and various other complexing agents in order to minimise the inhibitory effect of phytate on iron absorption.

Clearly, some of the classical pretreatments such as soaking, malting and germination in fact serve to activate the endogenous cereal phytases, and in turn become phytase-mediated approaches.

Several approaches involving genetically modified crops, either with low phytate content or higher phytase activity, have also been considered [2], but these strategies have not yet gained ground.

Finally, direct fortification of foods and separate iron supplementation are of course still potential solutions to improve the iron status of vulnerable populations. As already discussed above, iron supplementation may work in combination with phytase-mediated degradation of phytate for improving iron absorption from phytate-rich meals.

## 12. Conclusions

Several studies indicate that microbial phytase ingested with a phytate-rich meal (with the phytase added to the food) or added as a pretreatment aid has potential for increasing iron absorption from single foods. The action mechanism is enzymatic phytate degradation, which—if virtually complete—will result in release of chelated iron. It seems favourable to include citric and/or ascorbic acid in meals as these have been shown convincingly to act synergistically with the phytases in increasing iron absorption. The improved iron absorption potentially improves iron status in particularly iron-deficient individuals.

To our knowledge, only the *A. niger* phytase enzyme has been used in *in vivo* studies. It is therefore tempting to conclude that this particular enzyme is particularly effective in improving iron absorption via phytase degradation, but *in vitro* studies have suggested that other phytases can also effectively catalyse the degradation of phytate under physiologically relevant conditions. Fungal phytases have higher thermal stability than bacterial phytases, but the currently available data suggests that bacterial phytases, e.g., *E. coli* phytase, may also have potential due to an apparently higher proteolytic resistance against pepsin compared with the *A. niger* phytase, which in turn is more stable than the wheat phytase. Options for improving phytase stability in the gut should be investigated, including genetic engineering and/or formulation approaches. There are no substitutes for *in vivo* studies, but Caco-2 cell uptake studies have given good pre-clinical results and may provide a means to better unravel the detailed mechanisms of cellular iron receptor activation, compare iron bioavailability from different foods as well as deduce any correlations between phytase action rates for degradation of phytate, iron release, and cellular absorption rates. In any case, to improve our quantitative understanding the research must emphasise analysis and reporting of remaining inositol phosphate contents as well as phytate:iron ratios in the final digesta/pretreated food, since these parameters are crucial for determining iron absorption *in vivo*.

Further research on the exact chemical form of iron and phytate in the stomach and especially the possibility of cellular uptake from iron loosely chelated to lower inositol phosphates is needed in order to elucidate further the potential and mechanism of phytase-mediated iron release and absorption in the human body. In relation to this, a better understanding of the affinity of phytases towards soluble and insoluble iron phytate complexes present in phytate globoids is warranted. A deeper understanding of the significance of phytase dosage, kinetics (*i.e.*, rates of dephosphorylation), and stability under gastrointestinal conditions would aid in identifying key focus points with regard to design of intelligent solutions for *in vivo* phytase catalysis. These issues are crucial for defining the efficacy of phytase-mediated iron release, as the time window for *in vivo* catalysis will be limited by both enzyme stability and gastrointestinal transit times. With more knowledge on these issues, engineering of better dietary solutions for improved iron absorption from cereal and other plant foods would be possible.
